# Therapeutic Effects of Citrus Flavonoids Neohesperidin, Hesperidin and Its Aglycone, Hesperetin on Bone Health

**DOI:** 10.3390/biom12050626

**Published:** 2022-04-23

**Authors:** Adriana de Cássia Ortiz, Simone Ortiz Moura Fideles, Carlos Henrique Bertoni Reis, Márcia Zilioli Bellini, Eliana de Souza Bastos Mazuqueli Pereira, João Paulo Galletti Pilon, Miguel Ângelo de Marchi, Cláudia Rucco Penteado Detregiachi, Uri Adrian Prync Flato, Beatriz Flavia de Moraes Trazzi, Bruna Trazzi Pagani, José Burgos Ponce, Taiane Priscila Gardizani, Fulvia de Souza Veronez, Daniela Vieira Buchaim, Rogerio Leone Buchaim

**Affiliations:** 1 Department of Biological Sciences, Bauru School of Dentistry (FOB/USP), University of São Paulo, Bauru 17012-901, Brazil; adrianaortiz@usp.br (A.d.C.O.); simoneortiz@usp.br (S.O.M.F.); dr.carloshenriquereis@usp.br (C.H.B.R.); 2 UNIMAR Beneficent Hospital (HBU), Faculty of Medicine, University of Marilia (UNIMAR), Marília 17525-160, Brazil; joao.pilon@abhu.com.br (J.P.G.P.); uriflato@unimar.br (U.A.P.F.); 3 Pro-Rectory of Research and Graduate Studies, University Center of Adamantina (UniFAI), Adamantina 17800-000, Brazil; mzbellini@fai.com.br; 4 Postgraduate Program in Structural and Functional Interactions in Rehabilitation, Postgraduate Department, University of Marilia (UNIMAR), Marília 17525-902, Brazil; elianabastos@unimar.br (E.d.S.B.M.P.); claurucco@unimar.br (C.R.P.D.); danibuchaim@alumni.usp.br (D.V.B.); 5 Postgraduate Program in Speech Therapy, Sao Paulo State University (UNESP—Univ Estadual Paulista), Marília 17525-900, Brazil; 6 Coordination of the Medical School, University Center of Adamantina (UniFAI), Adamantina 17800-000, Brazil; coordmedicina@fai.com.br; 7 Coordination of the Dentistry School, University of Marilia (UNIMAR), Marília 17525-902, Brazil; flavia.odonto@unimar.br; 8 Dentistry School, University of Marilia (UNIMAR), Marília 17525-902, Brazil; brutrazzi@unimar.br; 9 Medicine Department, University Center of Adamantina (UniFAI), Adamantina 17800-000, Brazil; pepeburgos@fai.com.br (J.B.P.); taiane@fai.com.br (T.P.G.); 10 Medicine Department, Faculties of Dracena (FUNDEC Unifadra), Dracena 17900-000, Brazil; 11 Faculty of Medicine São Leopoldo Mandic (SLMANDIC), Araras 13606-134, Brazil; 12 Pro-Rectory of Teaching, University Center of Adamantina (UniFAI), Adamantina 17800-000, Brazil; fulveronez@fai.com.br; 13 Teaching and Research Coordination of the Medical School, University Center of Adamantina (UniFAI), Adamantina 17800-000, Brazil; 14 Graduate Program in Anatomy of Domestic and Wild Animals, Faculty of Veterinary Medicine and Animal Science, University of São Paulo (FMVZ/USP), São Paulo 05508-270, Brazil

**Keywords:** flavonoids, bone, bone repair, bone health, neohesperidin, hesperidin, hesperetin

## Abstract

Flavonoids are natural phytochemicals that have therapeutic effects and act in the prevention of several pathologies. These phytochemicals can be found in seeds, grains, tea, coffee, wine, chocolate, cocoa, vegetables and, mainly, in citrus fruits. Neohesperidin, hesperidin and hesperetin are citrus flavonoids from the flavanones subclass that have anti-inflammatory and antioxidant potential. Neohesperidin, in the form of neohesperidin dihydrochalcone (NHDC), also has dietary properties as a sweetener. In general, these flavanones have been investigated as a strategy to control bone diseases, such as osteoporosis and osteoarthritis. In this literature review, we compiled studies that investigated the effects of neohesperidin, hesperidin and its aglycone, hesperetin, on bone health. In vitro studies showed that these flavanones exerted an antiosteoclastic and anti- inflammatory effects, inhibiting the expression of osteoclastic markers and reducing the levels of reactive oxygen species, proinflammatory cytokines and matrix metalloproteinase levels. Similarly, such studies favored the osteogenic potential of preosteoblastic cells and induced the overexpression of osteogenic markers. In vivo, these flavanones favored the regeneration of bone defects and minimized inflammation in arthritis- and periodontitis-induced models. Additionally, they exerted a significant anticatabolic effect in ovariectomy models, reducing trabecular bone loss and increasing bone mineral density. Although research should advance to the clinical field, these flavanones may have therapeutic potential for controlling the progression of metabolic, autoimmune or inflammatory bone diseases.

## 1. Introduction

Bone is a highly resistant tissue composed of a mineralized organic matrix and different types of cells [1,2]. Despite considerable mechanical resistance, bone can be damaged by trauma, pathologies and osteometabolic disorders, which increase tissue susceptibility to fracture. However, bone tissue is in a constant process of remodeling, so it has considerable regenerative capacity [2,3]. Considering the active metabolism of bone, the integrity of this tissue depends on the balance between the processes of bone formation and resorption [1,4].

**Figure 1:**
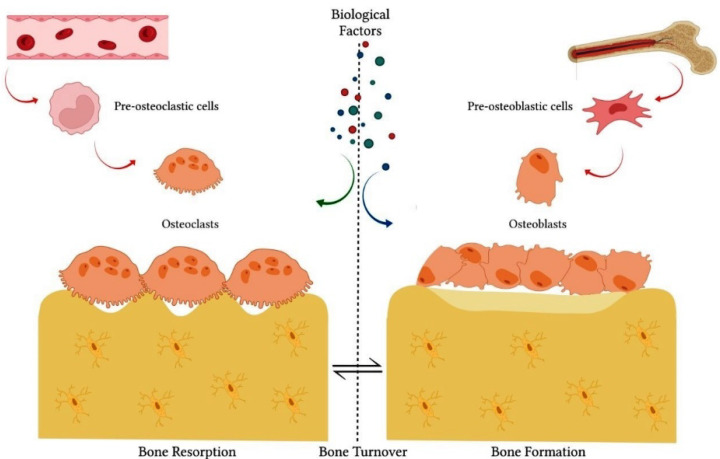
The integrity of bone tissue depends on the balance between bone formation and bone resorption processes. These processes are coordinated by the interactive action of specialized cells, such as osteoblasts and osteoclasts. Several biological factors also act in the process of physiological bone remodeling, such as cytokines, hormones, growth factors, transcriptional factors, ions and proteins of the extracellular matrix, in addition to other cell lines.

The balance between bone formation and resorption processes is coordinated by the interaction of several factors that act in the microenvironment, such as bone cells, cytokines, hormones, growth factors, transcriptional factors, ions and proteins of the extracellular matrix [2,3]. In the bone tissue environment, specialized cells, such as osteoblasts and osteoclasts, are responsible for bone matrix secretion and resorption, respectively [2,3] (Figure 1). Proteins synthesized by osteoblasts, such as alkaline phosphatase (ALP) and osteocalcin (OCN), are directly involved in matrix mineralization [5,6]. Several transcription factors, such as runt-related transcription factor 2 (Runx2) and Osterix (OSX), constitute key factors for the osteoblastic differentiation of undifferentiated cells, such as mesenchymal stem cells (MSCs) [6,7]. Likewise, bone morphogenetic proteins (BMPs) and Wnt/β-catenin signaling are the main pathways responsible for modulation of transcription factors related to osteoblastic differentiation [6,7]. In turn, osteoclasts regulate the process of bone formation and promote tissue resorption by the action of acids and proteolytic enzymes secreted into the bone matrix [8].

The main classes of enzymes that act on the degradation of the organic matrix are cysteine proteases and matrix metalloproteinases (MMPs) [9]. Osteoclastogenesis is also crucially regulated by several factors, such as receptor activator of nuclear factor-κB ligand (RANKL), receptor activator of nuclear factor-κB (RANK), osteoprotegerin (OPG) and monocyte colony-stimulating factor (M-CSF) [8]. M-CSF constitutes a hematopoietic growth factor that promotes the proliferation and differentiation of osteoclast progenitors, such as monocyte/macrophage-lineage cells [8,10]. RANKL and OPG are proteins expressed predominantly by osteoblasts and play an important role in bone formation. RANKL binds to RANK present on osteoclasts and their precursors [8]. RANKL/RANK interaction promotes osteoclast differentiation, activity and survival [8,11]. In turn, OPG acts as a competitive inhibitor by interacting with RANKL [8]. Thus, the RANKL/OPG ratio and crosstalk between osteoblasts and osteoclasts are directly related to bone turnover [8,11,12,13].

However, bone metabolism can be affected by several factors, such as age and pathophysiological conditions of the organism. Bone loss associated with advance of age is due to and imbalance between osteoblast and osteoclast activities [8,13]. Estrogen deficiencies are commonly associated with increased bone loss, as estrogen regulates osteoclast activity and apoptosis [13]. Likewise, hormonal changes and metabolic disorders can accelerate the resorption process and cause bone diseases, such as osteoporosis, characterized by reduced bone mass [14]. Metabolic disorders and autoimmune diseases, such as rheumatoid arthritis, are often associated with inflammatory reactions [15]. Consequently, inflammatory processes and immune reactions can disrupt bone homeostasis and accelerate tissue resorption. Under these conditions, increased levels of inflammatory cytokines, such as tumor necrosis factor-alpha (TNF-α), interleukin-1 (IL-1), interleukin-6 (IL-6), interleukin-7 (IL-7) and interleukin-17 (IL-17), stimulate the expression of RANKL, consequently favoring osteoclastogenesis [13]. Additionally, cellular oxidative stress promotes an increase in the production of reactive oxygen species (ROS) in bone cells. In turn, increased levels of ROS favor bone resorption by stimulating osteoblast apoptosis and senescence, RANKL overexpression and osteoclastic differentiation [13,16]. Therefore, bone turnover is influenced by several biological conditions.

Despite the native biological mechanisms that contribute to tissue healing, in many situations, therapeutic interventions are necessary to favor bone tissue regeneration. Some therapies have been proposed to promote the regeneration of extensive injuries and to improve the biomechanical properties of newformed bone; such proposed therapies include bone grafts [17,18], biomaterials [19,20,21,22], growth factors [18,23], photobiomodulation or ultrasound strategies [24,25,26] and cell-based therapies [27,28,29]. Furthermore, considering that the age and pathophysiological conditions of the organism can also accelerate bone loss, other therapeutic strategies have been investigated to minimize bone loss or to induce bone neoformation. Among them, the use of natural phytochemical agents has been highlighted as a promising therapeutic proposal, considering that these natural compounds have several beneficial biological properties. It has been reported that nutritional phytochemicals present in fruits, vegetables and cereals, such as flavonoid polyphenolic compounds, can improve bone mineral density by several mechanisms, in addition to having beneficial effects on bone homeostasis and health [13,16]. In addition to their nutritional properties, flavonoids may have applicability in the manufacture of polymers and can be used as stabilizing agents for biopolyesters, such as polylactide (PLA) and polyhydroxyalkanoate (PHA), which have been widely used in tissue engineering for membranes and scaffolds [30,31]. Thus, in this literature review, we focused on the biological effects on bone tissue health of three citrus flavonoids from the flavanones subclass: neohesperidin, hesperidin and its aglycone, hesperetin.

### 1.1. Biological Properties of Flavonoids

**Figure 2:**
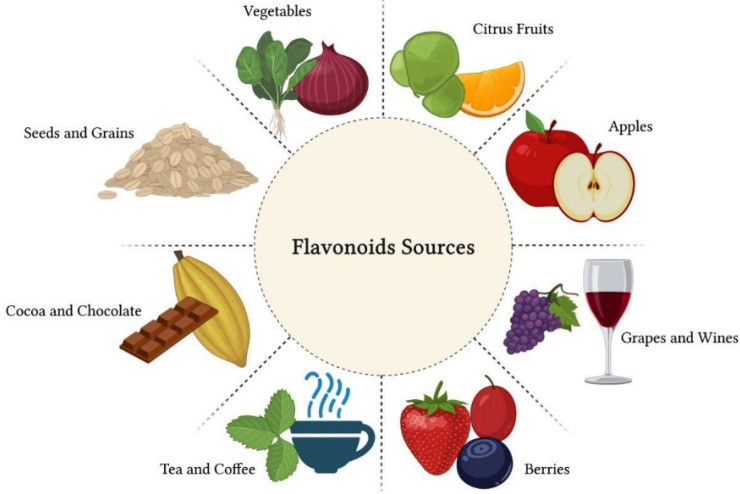
Flavonoids are polyphenolic compounds present in citrus fruits, grapes, raspberries, apples, vegetables, legumes and grains, as well as beverages, such as green tea, cocoa, coffee and red wine. These natural compounds have great nutritional value due to their therapeutic properties.

Flavonoids are polyphenolic compounds present in citrus fruits, grapes, raspberries, apples, vegetables, legumes and grains, as well as beverages, such as green tea, cocoa, coffee and red wine [32] (Figure 2). Structurally, flavonoids are formed by a carbon chain and can be classified into several subgroups according to the chemical composition of their structure. The main flavonoid subgroups of nutritional interest are flavanols or catechins, flavones, flavonols, flavanones, anthocyanidins and isoflavones [33].

In general, flavonoids have therapeutic properties and can provide several health benefits. The therapeutic effect of flavonoids is attributed to antioxidant, anti-inflammatory, antiallergic, antimicrobial, antitumor and antiviral properties [34,35,36]. There is evidence in the literature that regular consumption of a diet rich in flavonoids can contribute to the prevention of cardiovascular, neurodegenerative and inflammatory diseases, as well as metabolic disorders, such as diabetes mellitus [33,37,38]. Some studies have reported that flavonoids can reduce plasma levels of triglycerides and cholesterol and act by inhibiting tumor growth [39,40]. Other studies have shown that flavonoids can reduce the formation of ROS and the synthesis of inflammatory cytokines [38,41,42]. Comalada et al. (2006) reported that flavonoids, including the flavanone hesperetin, significantly inhibited the proliferation of bone marrow macrophages in vitro without significantly reducing the viability of these cells [42]. In the same study, some flavonoids were found to downregulate inducible nitric oxide synthase (iNOS) expression and nitric oxide (NO) production in cultures of LPS-activated macrophages, in addition to inhibiting the release of TNF-α by these cells [42].

**Figure 3:**
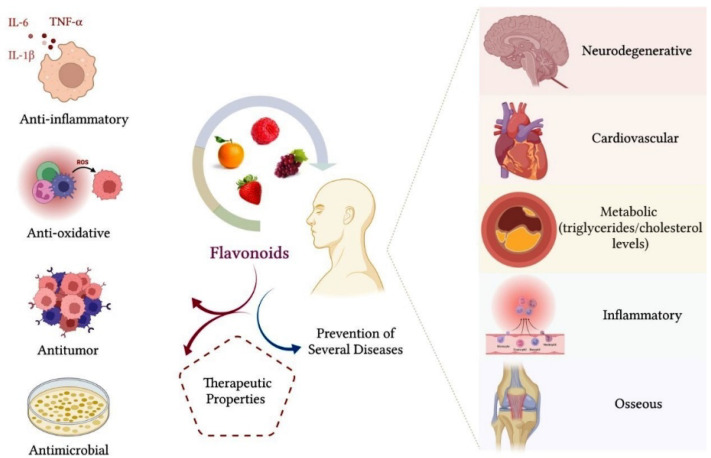
Flavonoids have an important therapeutic effect on the organism. The main benefits that flavonoids provide for health are related to their anti-inflammatory, antioxidant, antitumor and antimicrobial properties. Consumption of a diet containing flavonoids can also favor a reduction in triglycerides and cholesterol plasma levels, in addition to contributing to the prevention of neurodegenerative, cardiovascular, metabolic, inflammatory and bone diseases, such as osteoarthritis and osteoporosis.

Hwang and Yen (2008) showed that flavonoids inhibited ROS formation in vitro, significantly reducing cell oxidative stress [38]. The control of cellular oxidative stress is essential for tissue protection, considering that ROS react with macromolecules, such as proteins, carbohydrates, lipids and nucleic acids, which can cause irreversible deleterious changes [43]. Cheng et al. (2017) evaluated the anti-inflammatory properties of Citrus wilsonii Tanaka extract, a traditional Chinese medicine used to treat cough and sputum [41]. Citrus wilsonii Tanaka extract contains large amounts of naringin, as well as other flavonoids, such as naringin, eriocitrin, hesperidin, neohesperidin, rhoifolin, naringenin and poncyrin. In addition to showing very low cytotoxicity in macrophage culture, this extract significantly inhibited the synthesis of prostaglandins E2 (PGE2) and cyclooxygenase-2 (COX-2) and suppressed the expression of inflammatory mediators, such as TNF-α, IL-6 and interleukin-1 beta (IL-1β) [41]. The authors reported that the flavonoids present in the extract may have considerable potential for the treatment of chronic inflammatory diseases [41]. Figure 3 summarizes the therapeutic properties of flavonoids.

The benefits of flavonoid intake also extend to bone tissue health. Some flavonoids, such as isoflavones, are particularly classified as phytoestrogens because they are able to bind to estrogen receptors and have a similar action to that of this hormone, favoring bone anabolism [16]. Due to their anti-inflammatory, antioxidant and antiapoptotic properties, flavonoids have a beneficial effect on bone cell metabolism, contributing to the prevention of bone diseases, such as osteoarthritis and osteoporosis [44]. In bone, the inhibitory effect of flavonoids on oxidative stress and the inflammatory process favors the survival of osteoblasts and modulates osteoclastic differentiation.

Flavonoids have an important anti-inflammatory potential, which is crucial to minimizing tissue damage. In general, flavonoids act by inhibiting the synthesis of important inflammatory mediators, such as TNF-α, IL-1, IL-6 and IL-7, which is extremely beneficial, considering that these cytokines stimulate osteoclastic activity. It is also reported that flavonoids exert a regulatory effect on osteoclastic activity by inhibiting the expression of markers involved in bone resorption, such as RANKL and proteolytic enzymes, including MMPs, cathepsin-K and tartrate-resistant acid phosphatase (TRAP) [12,44]. Additionally, there is evidence in the literature that flavonoids stimulate the expression of osteogenic markers related to osteoblastic differentiation and bone matrix mineralization, such as Runx2, ALP, OCN, type 1 collagen (COL-1), osteopontin (OPN) and morphogenetic protein-2 (BMP-2) [13,16,44]. Flavonoids can also stimulate osteogenic differentiation of MSCs and preosteoblastic cells through the activation of important pathways, such as Smad1/5/8 and Wnt/β-catenin signaling pathways [13,16,44]. Through these mechanisms of action, flavonoids may favor bone formation and increase bone mineral density [13].

Among the different subclasses of flavonoids, in this review, we compiled studies that investigated the osteoprotective effects of the citrus flavonoids neohesperidin, hesperidin and its aglycone form, hesperetin, belonging to the flavanones subclass. These flavanones are found mainly in citrus fruits; like other flavonoids, they have important biological properties [32]. Neohesperidin (hesperetin-7-neohesperidoside) has considerable antioxidant, anti-inflammatory and antiapoptotic effects, in addition to dietary properties [34,45]. Hesperidin (hesperetin-7-O-rutinoside) and its aglycone, hesperetin, have a protective effect against bone loss, in addition to other properties, such as anti-inflammatory, antioxidant, anticancer and antimicrobial effects, as well as cardioprotective and anticholesterolemic activities [46,47,48,49,50,51]. There are also reports that hesperidin and hesperetin have a beneficial effect on neurological diseases and metabolic disorders, such as diabetes mellitus [49].

### 1.2. Neohesperidin, a Citrus Flavanone with Dietary Properties

Neohesperidin has great utility in the food industry and is widely used as a natural source for the synthesis of neohesperidin dihydrochalcone (NHDC), a low-calorie semisynthetic sweetener with intense sweetness [34,45]. The sweetening power of NHDC is approximately 1500 times greater than that of sucrose [52], and its caloric value does not exceed 0.002 kcal/g [53]. As with other flavonoids, NHDC is largely metabolized by the intestinal microflora [32], and like the natural form of neohesperidin, NHDC may have a beneficial therapeutic effect. According to Shi et al. (2015), NHDC has a protective effect on cellular metabolism [54]. The likely mechanisms by which this process occurs are due to both the inhibition of the nuclear factor-κB (NF-κB) signaling pathway and the synthesis of inflammatory cytokines, such as IL-6, IL-1β and TNF-α [54]. Considering the potential benefit of NHDC for health, in vitro and animal model studies have been conducted to evaluate possible mutagenic potential or to assess the safety of the administration of this sweetener through diet. The data obtained from these studies showed no evidence of carcinogenic or teratogenic effects that could be associated with NHDC, and no significant toxicological effects were reported in animals treated with high doses of this sweetener [54,55,56,57,58].

Lina et al. (1990) performed a study to identify possible adverse effects resulting from the daily administration of a diet containing NHDC [56]. The results of this study showed that the animals in the experimental group exhibited no hematological or histopathological changes after 91 days of treatment with NHDC [56]. Considering the negative toxicological tests and dietary properties, NHDC was recognized as a GRAS (generally recognized as safe) flavoring ingredient, and neohesperidin in the form of NHDC has been used as a non-nutritive sweetener in several food formulations, soft drinks and chewing gum. Sweeteners are natural or synthetic substances that have a much higher sweetening power than sucrose and can be used in smaller amounts. According to the Food and Drug Administration (FDA), non-nutritive sweeteners include sweeteners that have less than 2% of the caloric value of sucrose (4 kcal/g).

Thus, the search for sucrose substitutes has increased remarkably in recent decades, considering that several systemic pathologies and metabolic disorders, such as obesity and diabetes mellitus, have been associated with excessive consumption of caloric sugars [53]. Thus, various sucrose substitutes, such as cyclamate, saccharin, aspartame, neohesperidin (NHDC) and several others, have been used as sweeteners, providing an intense sweet taste when added to foods. In addition to the wide applicability of neohesperidin in the food industry, this flavanone can also be used as a non-nutritive agent in several other applications. In the pharmaceutical industry, neohesperidin can be used in cosmetic and dental products, such as toothpastes and oral care products [56].

### 1.3. Effect of Citrus Flavanones on Mineralized Tissues

In addition to the general health benefits, the use of flavonoids contributes to maintaining the integrity of the mineralized tissues of the organism, such as dental and bone tissues. Regarding dental tissues, there are reports in the literature that flavonoids have an anticollagenolytic effect and that some of these agents, such as neohesperidin, may have a probable antimicrobial action. Studies have shown that flavonoids act by inhibiting the expression of MMPs, which play an important role in the processes of remodeling and degradation of the extracellular matrix [37,39,59,60,61]. MMPs constitute a family of endopeptidases, which are zinc and calcium-binding enzymes present in biological tissues, such as saliva, gingival crevicular fluid and dentin [37]. MMPs have collagenolytic activity, and they are involved in several pathological disorders. In dentin, MMPs can be activated by the products of biofilm metabolism, caries and bacterial proteases. The activation of MMPs favors the degradation of collagen fibers, accelerating the process of dentin caries [62,63].

The effect of flavonoids on the dentin matrix has been reported in several studies. Ito et al. (1999) showed, in vitro, that flavonoids inhibited the synthesis of MMPs-1, 3, and 9, in addition to PGE2 [59]. Corroborating these data, later studies reported that flavonoids inhibited the proteolytic activity of MMPs, acting as a natural collagen stabilizer [37,60,61]. pH cycling model studies showed that incubation of specimens with hesperidin preserved and stabilized dentin collagen, minimizing demineralization and favoring remineralization in deep lesions, even in the absence of fluoride [64,65]. Stript et al. (2015) investigated the anticollagenolytic activity of hesperidin in an in situ model. The authors reported that specimens treated with hesperidin showed a significant reduction in dentin matrix loss (24%) compared to the control group [61]. Liu et al. (2017) showed that demineralized dentin treated with flavonoids exhibited less loss of hydroxyproline and tissue structure [60]. These authors observed that flavonoids preserved the integrity of collagen fibers and conserved the architecture of the dentin matrix, exhibiting a dose-dependent effect [60].

**Figure 4:**
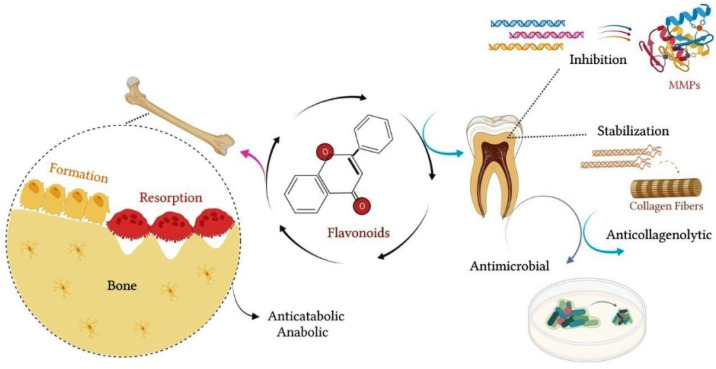
Flavonoids contribute to the maintenance of the integrity of mineralized tissues. In bone, flavonoids exert an anticatabolic effect, reducing the resorption process and favoring bone formation. In dental tissue, flavonoids have anticollagenolytic activity on the dentin matrix, inhibiting the proteolytic activity of MMPs and acting as a natural collagen stabilizer. In addition, flavonoids may exert an antimicrobial effect, inhibiting the proliferation of oral bacterial strains.

Other studies showed that hesperidin significantly reduced collagen degradation, providing greater tissue resistance to enzymatic degradation and reinforcing the mechanical properties of the dentin matrix [66,67]. According to these studies, pretreatment of dentin with adhesive agents containing flavonoids can minimize dentin biodegradation and increase the longevity of adhesive restorations [66,67]. Additionally, Manconi et al. (2018) investigated the anti-oxidant and anti-microbial effects of the nanoincorporation of flavonoid extract containing neohesperidin through the introduction of lipo- and glycerosomal vesicles in keratinocyte cultures and in planktonic cultures of microorganisms [68]. These authors showed that this extract reduced oxidative damage and keratinocyte apoptosis [68]. In the same study, the flavonoid extract containing neohesperidin inhibited the proliferation of S. mutans, S. sanguinis and Lactobacillus, suggesting a probable antimicrobial effect [68]. Figure 4 schematizes the main effects of flavonoids on mineralized tissues.

### 1.4. Effects of Citrus Flavanones on Bone Tissue

#### 1.4.1. Neohesperidin

In bone tissue, citrus flavanones have a beneficial effect on osteogenic cells and their functions. Recent studies have shown that neohesperidin can inhibit the expression of osteoclastic differentiation markers, in addition to inducing the expression of genes related to osteoblastic differentiation and matrix mineralization. With in vitro and in vivo studies, Tan et al. (2017) showed that neohesperidin exerted an inhibitory effect on osteoclastic differentiation and bone resorption, [69]. These authors reported that neohesperidin inhibited RANKL-induced osteoclast differentiation and the expression of cathepsin K and TRAP markers in osteoclastic cultures. In the same study, neohesperidin also suppressed the activity of the transcription factor NF-kB involved in the inflammatory response, cellular oxidative stress and the production of free radicals. Furthermore, neohesperidin reduced osteoclast calcium oscillation, indicating an inhibition of the RANKL-induced calcium signaling pathway during osteoclast differentiation [69]. In vivo, the administration of neohesperidin exerted an antiosteoclastic effect, protecting against bone loss in ovariectomized mice [69]. Results obtained by micro-CT showed that neohesperidin significantly reduced trabecular bone loss and achieve effects similar to those obtained by estrogen administration. Histomorphometric data showed a significant reduction in osteoclast number/bone surface (N.Oc/BS) and osteoclast surface/bone surface (Oc.S/BS) in the neohesperidin group [69]. These findings showed that neohesperidin exerted an anticatabolic effect in OVX mice, reducing the number and, consequently, biological activity of osteoclasts [69].

Chang et al. (2021) also reported that neohesperidin may favor the proliferation and osteoblastic differentiation of MSCs [70]. These authors showed that bone marrow MSCs treated with neohesperidin had 855 differentially expressed genes, with overexpression of genes related to the Wnt/β-catenin signaling pathway. In this study, neohesperidin significantly favored MSC proliferation, ALP activity, calcium deposition and the expression of osteogenic markers, such as Runx2, OCN, BMP-2 and β-catenin. However, the effects of neohesperidin were partially blocked by the addition of Dickkopf (DKK1), an antagonist of the Wnt/β-catenin pathway, suggesting that the activation of this signaling pathway may be one of the mechanisms of action of this flavonoid [70]. Corroborating these data, Zhang et al. (2021) reported that neohesperidin favored cell viability and osteogenic differentiation of MSCs, with a significant increase in the expression of Runx2, OCN and ALP markers [71]. In this study, neohesperidin also significantly increased alkaline phosphatase activity and calcium nodule deposition after 14 days of culture. Taken together, these data suggest that neohesperidin could be considered as a useful pharmacological agent for controlling the progression of osteoporosis and other disorders that affect bone tissue, such as rheumatoid arthritis [69,71].

Wang et al. (2021) investigated the therapeutic effect of neohesperidin on TNF-α-stimulated human rheumatoid arthritis fibroblast-like synoviocytes and found that neohesperidin may have therapeutic potential to control the progression of rheumatoid arthritis [72]. Rheumatoid arthritis is an autoimmune disease characterized by a chronic inflammatory process that affects the synovial membranes of joints. These authors showed that the treatment of cells with neohesperidin resulted in a decrease in the levels of proinflammatory cytokines IL-1β, IL-6, IL-8 and TNF-α, as well as metalloproteinases MMP-3, MMP-9 and MMP-13, without compromising cell viability. Neohesperidin also reduced the accumulation of ROS caused by oxidative stress induced by TNF-α. Analysis by qRT-PCR further revealed that OPG was upregulated, whereas RANKL was downregulated in fibroblast-like synoviocytes treated with neohesperidin [72]. According to these authors, the effects of neohesperidin on fibroblast-like synoviocytes may be partially due to the inhibition of the MAPK signaling pathway [72].

The balance between bone formation and resorption is also positively influenced by the anti-inflammatory potential of neohesperidin and flavonoids in general, which act by inhibiting the synthesis of pro-inflammatory cytokines and the production of ROS. Choi et al. (2021) investigated the anti-inflammatory potential of the sweetener neohesperidin dihydrochalcone (NHDC) and its natural metabolite, dihydrocaffeic acid (DHCA), in macrophage and adipocyte cultures [73]. These authors showed that NHDC and DHCA exerted an anti-inflammatory effect on macrophages and adipocytes, leading to a significant reduction in the production of TNF-α and IL-6 cytokines. In this in vitro study, NHDC also interfered with the lipid metabolism of adipocytes through its metabolite, DHCA. Thus, NHDC significantly increased adipocyte fat deposition, which was considerably inhibited by its metabolite, DHCA [73]. The anti-inflammatory effects of NHDC were also observed in mice with high-fat-diet-induced obesity (HFD) [73]. Ad libitum intake of NHDC significantly reduced weight gain in HFD mice. Additionally, M2-polarized, bone-marrow-derived macrophages from HFD mice fed NHDC showed an increased secretion of the anti-inflammatory cytokine IL-10, suggesting a beneficial effect of NHDC on the inflammatory process associated with obesity [73]. In summary, NHDC and DHCA showed anti-inflammatory potential in vitro and in vivo, improving the physiological condition related to obesity [73].

Table 1 summarizes the main outcomes of in vitro and in vivo studies that evaluated the osteoprotective and anti-inflammatory effects of neohesperidin or neohesperidin dihydrochalcone (NHDC)/dihydrocaffeic acid (DHCA).

#### 1.4.2. Hesperidin

The biological properties of hesperidin and its aglycone form, hesperetin, on bone tissue have also been reported in several studies. Studies using animal models have shown that hesperidin can minimize bone loss and favor the regeneration of bone defects. In addition, there is evidence that hesperidin has anti-inflammatory and anticancer properties. Chiba et al. (2003) reported that the administration of a diet containing hesperidin significantly inhibited bone loss in OVX mice, with a beneficial effect on morphometric parameters, such as trabecular bone volume and thickness [75]. In this study, analyses of femur mineral content also showed that hesperidin-OVX and estrogen-OVX mouse groups had similar concentrations of Ca, P and Zn, significantly differing from the OVX group. Hesperidin also reduced the number of osteoclasts in the femoral metaphysis in similar proportions to the estrogen-OVX mice. Additionally, hesperidin-OVX mice showed lower levels of serum and liver lipids compared to OVX mice fed a control diet. According to these authors, the effect of hesperidin on bone and lipid metabolism may be useful in the prevention and control of diseases related to sedentary lifestyle and obesity [75]. Corroborating these data, Horcajada et al. (2008) also showed a protective effect of hesperidin on bone loss in OVX rats, accompanied by a lipid-lowering action [76].

Young (3 months) and adult (6 months) rats, subdivided into intact (sham) or ovariectomized (OVX) and fed a diet containing hesperidin, showed significant changes in bone metabolism. After 90 days, bone loss was completely inhibited in young OVX rats and partially inhibited in adult OVX rats, among which osteopenia tends to be more severe. Additionally, in the sham groups, the ingestion of hesperidin promoted a significant increase in bone mineral density in young rats and an improvement in bone strength in adult rats. In addition, hesperidin ingestion reduced the urinary excretion of deoxypyridinoline, a marker of bone resorption mediated by osteoclastic activity [76]. Deoxypyridinoline is present in the collagen matrix of both bone and dentin and is released during the resorptive process. These findings indicate that one of the mechanisms by which hesperidin acts to reduce bone loss is through the inhibition of bone resorption. Importantly, the benefits of hesperidin ingestion in bone metabolism were seen in both OVX and intact rats, suggesting that hesperidin constitutes an interesting option for consumption as a dietary ingredient [76].

In addition to the protective effect against ovariectomy-induced bone loss, hesperidin may have therapeutic potential in the control of arthritis. Umar et al. (2013) reported that hesperidin had the potential to inhibit collagen-induced arthritis in rats [77]. In this study, biochemical and histological analyses showed that hesperidin minimized the deleterious effects on bones and joints caused by rheumatoid arthritis, suppressing the progression of the disease. Treatment with hesperidin significantly reduced neutrophil activation and infiltration, which contributed considerably to the restoration of histological changes. According to these authors, the beneficial effect of hesperidin was attributed to the reduction in catalase, nitric oxide and free radical levels [77]. The anti-inflammatory potential of hesperidin was also reported by Kuo et al. (2018) [78]. In this study, ligature-induced alveolar bone loss in rats was significantly inhibited by intragastric administration of hesperidin. Animals fed hesperidin also had less gingival inflammation and reduced connective tissue loss. Gene expression analysis showed that hesperidin downregulated the expression of inflammatory markers, such as IL-6, IL-1β and iNOS. IL-6 and IL-1β are important pro-inflammatory cytokines, and iNOS plays a crucial role in osteoclastic activation and alveolar bone loss. These authors reported that hesperidin may constitute a promising agent to minimize inflammatory processes caused by oral diseases [78].

Considering that hesperidin has beneficial pharmacological properties for the treatment of various types of diseases, the administration of this flavonoid through a drug delivery system can also constitute an effective strategy to achieve certain therapeutic targets. Sulaiman et al. (2020) exposed a human breast cancer cell line to treatment with hesperidin loaded on gold nanoparticles and obtained a significant reduction in proliferation and inhibition of the growth of these cells [79]. In vivo experiments showed that mice treated with hesperidin loaded on gold nanoparticles showed no signs of toxicity. Analysis of internal organs, such as liver, spleen, lung and kidney, showed no histological or histopathological changes. Additionally, treatment with hesperidin loaded on gold nanoparticles potentiated the biological activity of macrophages in mice bearing Ehrlich ascites tumor cells and inhibited the synthesis of pro-inflammatory cytokines by bone marrow-derived macrophages, such as IL-1β, IL-6 and TNF-α [79]. The anti-inflammatory properties and osteoprotective effect of hesperidin were also demonstrated by Zhang et al. (2021) [71]. These authors used an ovariectomy-induced osteoporosis model in rats and showed that oral administration of hesperidin significantly increased bone mineral density, improved biomechanical parameters and considerably reduced levels of pro-inflammatory cytokines, such as IL-6, IL-1β and TNF-α. Hesperidin also significantly reduced the levels of bone turnover markers, such as ALP, OCN and acid phosphatase (ACP), in OVX rats [71].

There are also reports in the literature that hesperidin may have the potential to induce osteogenesis and to favor the regeneration of injured bone tissue. A recent study that evaluated the effects of hesperidin on viability, cell differentiation, collagen matrix secretion and mineralization in cultures of MC3T3-E1 lineage preosteoblastic cells showed that this flavonoid favored the osteogenic potential of these cells [80]. In addition to not affecting cell viability, the treatment of these cells with hesperidin resulted in the overexpression of osteogenic markers, such as Runx2, OSX, bone sialoprotein (BSP) and collagen type I alpha 2 chain (COL1A2). Hesperidin also favored the deposition, organization and maturation of the collagen matrix [80]. In addition to in vitro data, Miguez et al. (2021) also obtained promising results with the use of hesperidin in tissue engineering [80]. In a critically sized defect rat mandible model, implantation of a collagen sponge loaded with hesperidin and a suboptimal dose of BMP-2 induced significant bone formation in the defect (5 mm) after 2 weeks. Additionally, hesperidin promoted an increase in mature collagen fibers, with a beneficial effect on the maturation and organization of the organic matrix. Analyses by polarized light microscopy and micro-CT showed that hesperidin had positive effects on matrix mineralization and the quality of newly formed bone tissue. However, hesperidin alone was not sufficient to induce significant bone regeneration in mandibular defects [80]. Table 2 summarizes the main outcomes of in vitro and in vivo studies that evaluated the osteoprotective and anti-inflammatory effects of hesperidin.

#### 1.4.3. Hesperetin

Hesperetin, the aglycone form of hesperidin, also acts as a bioactive compound that may favor osteogenesis in vitro and in vivo. Trzeciakiewicz et al. (2010) reported that hesperetin favored osteoblastic activity in vitro and significantly increased alkaline phosphatase (ALP) activity, as well as the expression of osteogenic markers, such as BMP-2, BMP-4, Runx2 and OSX [81]. However, this osteogenic effect was blocked by the addition of the noggin inhibitor, suggesting that the action of hesperetin occurred through the signaling pathway of bone morphogenetic proteins [81]. Hesperetin metabolites can also influence the osteogenic potential of bone cells, regulating crosstalk between osteoblasts and osteoclasts. Trzeciakiewicz et al. (2010) reported that hesperetin-7-O-glucuronide significantly potentiated the osteogenic activity of osteoblasts in vitro [82]. Hesperetin-7-O-glucuronide is a metabolite conjugated to glucuronides, resulting from the hydrolysis of hesperidin by the intestinal microbiota. In osteoblasts from rat calvaria, hesperetin-7-O-glucuronide increased the phosphorylation of Smad1/5/8 signaling and ALP activity. Hesperetin-7-O-glucuronide also had effects on the expression of osteoclastic and osteogenic markers, downregulating RANKL and overexpressing ALP, Runx2 and OSX [82]. Hesperetin may also enhance the osteogenic potential of undifferentiated cells. Xue et al. (2017) reported that hesperetin promoted proliferation, migration and osteogenic differentiation of human MSCs (hMSCs) through increased phosphorylation of the ERK1/2 and Smad1/5/8 signaling pathways [83]. hMSCs treated with hesperetin showed overexpression of ALP, Runx2, OCN and COL1A1, with markedly expressive mineralization at 14 days of culture [83]. The osteogenic potential of hesperetin used as a growth factor in tissue engineering was also reported by these authors. In rat osteotomy models, hesperetin/gelatin sponge scaffolds loaded with hMSCs accelerated tibial fracture regeneration. Bone bridge formation in the defects was found in the hesperetin/gelatin hMSC and hesperetin/gelatin groups after 8 weeks postoperatively. Hesperetin was also able to inhibit RANKL-induced osteoclastogenesis without inducing cytotoxic effects [83].

The effects of hesperetin on bone cells and on trabecular bone architecture were also discussed by Zhang et al. (2018) [84]. Osteoclast precursor cells (RAW 264.7), spleen splenocytes and primary bone marrow monocytes (BMMs) from tibias and femurs were used to investigate the inhibitory effect of hesperetin on osteoclastogenesis in vitro. Additionally, a preosteoblastic cell line (MC3T3-E1) was used to assess the osteogenic differentiation potential of hesperetin. Zhang et al. (2018) reported that after RANKL stimulation, hesperetin significantly prevented the maturation of multinucleated osteoclasts in the three cell types, with a considerable decrease in the number and area of TRAP-positive osteoclasts. Likewise, the addition of hesperetin after RANKL stimulation also significantly impaired osteoclastic activity in vitro, decreasing resorption points and osteolytic lesions in bone slices, evaluated by scanning electron microscopy (SEM). In MC3T3-E1 cells, hesperetin increased the staining and activity of ALP, suggesting that it may have osteogenic potential in vitro [84]. In addition to impairing osteoclast differentiation and activity and favoring the osteogenic potential of preosteoblastic cells in vitro, hesperetin also minimized the deleterious effects of osteoporosis in vivo. In the OVX osteoporosis mouse model, intraperitoneal injections of hesperetin significantly improved histomorphometric parameters of trabecular bone, increasing the trabecular bone volume ratio (BV/TV), trabecular thickness (Tb.Th) and trabecular number (Tb.N) and decreasing trabecular separation (Tb.Sp). Additionally, hesperetin significantly decreased the number of TRAP-positive osteoclasts in tibial trabecular bone and increased ALP serum levels in OVX mice [84]. The effect of hesperetin on osteoclastic activity in vitro and on trabecular bone resorption in vivo was also reported by Liu et al. (2019) [85]. In this study, hesperetin significantly reduced the in vitro expression of osteoclastic markers, such as TRAP, MMP-9, cathepsin K, c-Fos and nuclear factor of activated T-cells cytoplasmic 1 (NFATC1). The authors attributed the inhibition of osteoclastogenesis by hesperetin to the elimination of reactive oxygen species and the inhibition of the NF-κB and MAPK signaling pathways [85]. Corroborating the in vitro findings, hesperetin reduced trabecular bone loss in mice with lipopolysaccharide (LPS)-induced osteoporosis. Mice treated with hesperetin showed a significant reduction in serological levels of RANKL, TNF-α, IL-1β and IL-6 inflammatory markers, whereas a considerable increase in OPG levels was observed. Consequently, the RANKL/OPG ratio was significantly decreased by the administration of hesperetin, resulting in less bone resorption [85].

An important issue to be considered is the fact that differences in the chemical structure of several flavonoids can influence metabolism, bioavailability and, consequently, the effects of these agents on bone tissue. Habauzit et al. (2009) conducted an in vivo study to investigate the relationship between plasma bioavailability and the protective potential of hesperidin and its aglycone, hesperetin-7-glucoside, on bone tissue [86]. In this study, OVX rats were fed a casein-based diet supplemented or not with freeze-dried orange juice containing hesperidin or hesperetin-7-glucoside. After 90 days, analysis of bone mineral density of the femurs showed that both flavonoids have the potential to minimize bone loss in OVX rats. However, hesperetin-7-glucoside showed a better osteoprotective effect, which may be related to the higher plasma availability of this flavonoid, as hesperetin-7-glucoside reached plasma levels twice as high as those of hesperidin [86]. Table 3 summarizes the main outcomes of in vitro and in vivo studies that evaluated the osteoprotective and anti-inflammatory effects of hesperetin and its metabolites.

### 1.5. Oral Bioavailability of Dietary Flavonoids

Although flavonoids present several biological properties, it has been reported that these compounds have low oral bioavailability due to their low solubility [87,88,89,90,91]. Flavonoids ingested through diet are metabolized by the gut microbiota and undergo extensive biotransformation [87]. In the diet, most flavonoids are present in the form of β-glycosides, which are bound to a sugar molecule [87,88]. The hydrolysis of β-glycosides and the cleavage of the sugar molecule result in their respective aglycone forms, which are easily absorbed [87]. In turn, aglycones can also be present in the diet but in smaller amounts [92]. Thus, the food industry has aimed to provide food supplements containing flavonoids in the form of aglycones [92], considering that the absorption rate and plasma concentration of flavonoids and their metabolites are crucial for these compounds to exert a therapeutic effect [89]. However, an important issue is the long process of elimination of these compounds; therefore, frequent consumption of a diet rich in flavonoids can have a beneficial effect on health [87].

Additionally, the literature also reports that the therapeutic properties of flavonoids are also due to their metabolites, which have greater bioavailability [89]. Several techniques have been developed to improve the solubility and absorption of these compounds, as well as to reduce their metabolic degradation. Among them, the structural modification of flavonoids has been highlighted as an effective technique to improve the bioavailability of these compounds [89,91]. In addition, technologies used by the pharmaceutical industry, such as nanotechnology and delivery systems, have also been developed to improve the oral bioavailability of flavonoids [90,91]. These technologies aim to allow the active forms of flavonoids to reach their therapeutic targets [91]. In general, despite their low oral bioavailability, flavonoid compounds exert a considerable therapeutic effect, showing high bioactivity [93].

## 2. Conclusions

Flavonoids are natural phytochemicals that may have therapeutic action, contributing to the prevention of several disorders. Neohesperidin, hesperidin and hesperetin are citrus flavonoids that have considerable anti-inflammatory and antioxidant potential. These flavanones also have a beneficial effect on mineralized tissues due to their anti-collagenolytic potential. In bone, in vitro studies showed that these flavanones exerted antiosteoclastic and anti-inflammatory effects, inhibiting the expression of osteoclastic markers (RANKL, cathepsin K and TRAP) and reducing the levels of ROS, proinflammatory cytokines (IL-1β, IL-6, IL-8 and TNF-α) and matrix metalloproteinases (MMP-3, MMP-9 and MMP-13).

Similarly, these flavanones favored the osteogenic potential of MSCs and preosteoblastic cells, promoting the overexpression of osteogenic markers, such as Runx2, ALP, OCN, BMP-2 and β-catenin. In vivo, these flavanones exerted a significant anticatabolic effect, reducing trabecular bone loss and increasing bone mineral density in ovariectomized animals. Likewise, they favored the regeneration of bone defects and reduced the inflammatory process in animals with arthritis or induced periodontitis models. These data indicate that the citrus flavanones neohesperidin, hesperidin and hesperetin may have therapeutic potential for controlling the progression of osteometabolic, autoimmune and inflammatory diseases, such as osteoporosis and osteoarthritis. However, research should advance to better investigate the effect of these phytochemicals, especially in clinical trials.

## Figures and Tables

**Table 1:** In vitro and in vivo studies that evaluated the osteoprotective and anti-inflammatory effects of neohesperidin or neohesperidin dihydrochalcone (NHDC)/dihydrocaffeic acid (DHCA).

Reference	In Vitro Models	Animals Models	Main Outcomes
Tan et al. (2017) [69]	Bone marrow macrophage-derived osteoclasts	Ovariectomized (OVX) mice	Neohesperidin exerted an anti-osteoclastic effect in vitro, suppressing the activity of the transcription factor NF-kB and inhibiting the expression of osteoclastic markers, such as RANKL, cathepsin K and TRAP. In OVX mice, neohesperidin exerted an anticatabolic effect, significantly reducing the number of osteoclasts and trabecular bone loss. Additionally, neohesperidin may have a protective effect on bone architecture, showing a bone volume/tissue volume ratio (% BV/TV) similar to that of the estrogen group.
Chang et al. (2021) [70]	Human bone marrow MSCs		Neohesperidin significantly favored the proliferation of MSCs, ALP activity, calcium deposition and the expression of osteogenic markers, such as Runx2, OCN, BMP-2 and β-catenin.
Choi et al. (2021) [73]	Murine macrophage cell line (RAW264.7)/pre-adipocytes (3T3-L1)	NHDC-fed mice	Neohesperidin dihydrochalcone (NHDC)/dihydrocaffeic acid (DHCA) exerted an anti-inflammatory effect on macrophage and adipocyte cultures, significantly reducing the levels of cytokines TNF-α and IL-6. NHDC intake significantly reduced weight gain in high-fat-diet-induced obesity mice (HFD) and increased secretion of the anti-inflammatory cytokine (IL-10) in M2-polarized, bone-marrow-derived macrophages from HFD mice.
Wang et al. (2021) [72]	Fibroblast-like synoviocytes		Neohesperidin reduced the levels of ROS, pro-inflammatory cytokines (IL-1β, IL-6, IL-8 and TNF-α) and metalloproteinases (MMP-3, MMP-9 and MMP-13) in human rheumatoid arthritis fibroblast-like synoviocytes. In addition, neohesperidin promoted the overexpression of OPG and the downregulation of RANKL.
Zhang et al. (2021) [74]	Human bone marrow MSCs		Neohesperidin significantly favored the osteogenic differentiation of MSCs, promoting ALP activity, calcium nodule deposition and expression of osteogenic markers, such as Runx2, OCN and ALP.

**Table 2:** In vitro and in vivo studies that evaluated the osteoprotective and anti-inflammatory effects of hesperidin.

Reference	In Vitro Models	Animals Models	Main Outcomes
Chiba et al. (2003) [75]		Ovariectomized (OVX) mice	Hesperidin significantly inhibited bone loss in OVX mice, with a beneficial effect on volume and thickness of femoral trabecular bone. In addition, hesperidin-OVX mice showed femur mineral content (Ca, P, Zn) similar to that of estrogen-OVX mice. Hesperidin also reduced the number of osteoclasts in the femoral metaphysis in similar proportions to those in the estrogen-treated group.
Horcajada et al. (2008) [76]		Young and adult ovariectomized (OVX) rats	Hesperidin showed a protective effect on bone loss in OVX rats, inhibiting bone loss completely in young OVX rats and partially in adult OVX rats. In the sham groups, hesperidin promoted a significant increase in bone mineral density in young rats and an improvement in bone strength in adult rats.
Umar et al. (2013) [77]		Collagen-induced arthritis rats	Hesperidin inhibited collagen-induced arthritis in rats, significantly reducing neutrophil activation and infiltration. Hesperidin reduced the levels of catalase, nitric oxide and free radicals, suppressing the progression of the disease.
Kuo et al. (2018) [78]		Rats with ligation-induced periodontitis	In rats, ligature-induced alveolar bone loss was significantly inhibited by intragastric administration of hesperidin. Additionally, hesperidin downregulated the expression of the inflammatory markers IL-6, IL-1β and iNOS. Hesperidin also reduced gingival inflammation and connective tissue loss.
Sulaiman et al. (2020) [79]	Human breast cancer cell line (MDA-MB-231)	Mice. Ehrlich ascites tumor cell-bearing mice	Hesperidin loaded on gold nanoparticles significantly inhibited the proliferation of human breast cancer cell line in vitro. No histopathological changes were found in mice treated with hesperidin. Hesperidin loaded on gold nanoparticles potentiated the biological activity of macrophages in mice with Ehrlich ascites tumor cells and inhibited the synthesis of proinflammatory cytokines (IL-1β, IL-6, TNF-α) by bone-marrow-derived macrophages.
Miguez et al. (2021) [80]	MC3T3-E1 pre-osteoblastic cells	Rat with critically sized mandible defect (5 mm)	Hesperidin favored the deposition/maturation of the collagen matrix in MC3T3-E1 lineage cultures and the overexpression of osteogenic markers, such as Runx2, OSX, BSP and COL1A2. In a critically sized defect rat mandible model, a collagen sponge loaded with hesperidin and a suboptimal dose of BMP-2 induced significant bone formation and favored organic matrix maturation and mineralization.
Zhang et al. (2021) [71]		Ovariectomy (OVX)-induced osteoporosis in rats	In OVX rats, oral administration of hesperidin significantly increased bone mineral density and considerably reduced levels of proinflammatory cytokines, such as IL-6, IL-1β and TNF-α. Additionally, hesperidin improved biomechanical parameters and significantly reduced the levels of bone turnover markers, such as ALP, OCN and ACP.

**Table 3:** In vitro and in vivo studies that evaluated the osteoprotective and anti-inflammatory effects of hesperetin and its metabolites.

Reference	In Vitro Models	Animals Models	Main Outcomes
Habauzit et al. (2009) [86]		Ovariectomized (OVX) rats	Hesperidin/hesperetin-7-glucoside: casein-based diets supplemented with freeze-dried orange juice containing hesperidin or hesperetin-7-glucoside were effective in promoting an increase in bone mineral density in OVX rats. However, hesperetin-7-glucoside has a higher plasma bioavailability than hesperidin (approximately two-fold higher), showing a better osteoprotective effect.
Trzeciakiewicz et al. (2010) [81]	Rat calvaria primary osteoblasts		Hesperetin favored osteoblastic differentiation and significantly increased ALP activity and expression of osteogenic markers, such as BMP-2, BMP-4, Runx2 and OSX.
Trzeciakiewicz et al. (2010) [82]	Rat calvaria primary osteoblasts		Hesperetin-7-O-glucuronide significantly increased ALP activity and promoted the overexpression of osteogenic markers, such as ALP, Runx2 and OSX. Additionally, hesperetin-7-O-glucuronide increased the phosphorylation of Smad1/5/8 signaling and downregulated RANKL expression.
Xue et al. (2017) [83]	Human MSCs	Rat tibial osteotomy model (1 mm)	Hesperetin favored the proliferation and migration of human MSCs in vitro. In addition, hesperetin favored osteogenic differentiation of human MSCs, increasing the phosphorylation of Smad1/5/8 signaling and promoting the overexpression of osteogenic markers, such as ALP, Runx2, OCN and COL1A1. In rat osteotomy models, hesperetin/gelatin and hesperetin/gelatin-hMSC groups presented significant bone formation. Hesperetin/gelatin sponge scaffolds loaded with hMSCs accelerated tibial fracture regeneration, resulting in complete fracture union without a cortical gap.
Zhang et al. (2018) [84]	Preosteoclastic cells (RAW 264.7); splenocytes; bone marrow monocytes (BMMs); Pre-osteoblastic cells (MC3T3-E1)	Ovariectomized (OVX) osteoporosis mouse model	RAW 264.7; Splenocytes; BMMs: Hesperetin significantly prevented the maturation of multinucleated osteoclasts in the three cell types after RANKL stimulation, with a considerable decrease in the number and area of TRAP-positive osteoclasts. Additionally, the addition of hesperetin also significantly impaired osteoclastic activity after RANKL stimulation, decreasing resorption points and osteolytic lesions in bone slices. MC3T3-E1: Hesperetin increased the staining and activity of ALP, suggesting that it may have osteogenic potential in vitro. In the OVX osteoporosis mouse model, intraperitoneal injections of hesperetin significantly improved histomorphometric parameters of trabecular bone, increasing BV/TV, Tb.Th and Tb.N and decreasing Tb.Sp. Additionally, hesperetin significantly decreased the number of TRAP-positive osteoclasts in tibial trabecular bone and increased ALP levels in OVX mice.
Liu et al. (2019) [85]	Murine macrophage cell line (RAW264.7)	Lipopolysaccharide-induced osteoporosis (LPS) in mice	Hesperetin significantly reduced the in vitro expression of osteoclastic markers, such as TRAP, MMP-9, cathepsin K, c-Fos and NFATC1. In vivo, hesperetin significantly reduced trabecular bone loss in LPS mice, with a significant reduction in serum levels of inflammatory markers (RANKL, TNF-α, IL-1β and IL-6) and a considerable increase in OPG levels.

## Data Availability

Not applicable.
